# The Treatment of Insomnia by Drugs

**Published:** 1902-06-14

**Authors:** 


### THE TREATMENT OF INSOMNIA BY DRUGS.

An interesting discussion on the use of narcotics in the treatment of sleeplessness was started by a paper by Dr. Henry Rayner at the last meeting of the Medico-Psychological Society. Dr. Rayner evidently greatly dislikes the employment of narcotics as a means of producing sleep, and he expressed the opinion that a considerable portion of the nervous and mental disorders met with in the well-to-do classes is due to the abuse of these drugs. Dr. G. F. Blandford, however, said he had been treating patients for 40 years, many of whom came to him in a state of threatening insanity, with insomnia as a very marked feature, and he felt as certain as he could feel of anything that a great number of these patients had been materially benefited by the use of medicines given to induce sleep. He held that it was possible to give drugs in such doses as to produce a state which was indistinguishable from natural sleep, but it was necessary to select the drug suitable to the case. Among the general body of the profession he thought that far too large doses of sedatives were given, and that they were continued for too long a time. One of the best and safest of narcotics was paraldehyde. Dr. Savage said that the symptom which he was consulted about more than any other was sleeplessness, and that in each case the cause of this condition must first of all be ascertained. In regard to anæmia of the brain, about which one heard a good deal, he was certain that a nightcap of grog with a little food were more likely to produce sleep than sedatives of the opium type. There were cases again in which hydrotherapy was of the utmost use—patients whose tissues were already loaded with toxic agents, who had taken too much alcohol, or were gouty. For such cases a dose of calomel followed by salines and baths was of great value in producing sleep as well as in curing the patient of his malady. In painful conditions opiates and other narcotics assisted in procuring sleep, and this was especially so in cases of mental pain, and though it was true that opium upset the digestion, the rest which it procured was the means of the digestion being in turn improved. In regard to the more extreme use of narcotics he said that he had seen extremely mischievous patients narcotised into dementia, and on recovery from that condition get perfectly well. A morphinomaniac again could be given an ounce of bromide per day for several days and be found to have recovered at the end of that time. He agreed with the statement made by Dr. Blandford to the effect that in many cases narcotics were of the greatest benefit in warding off threatened attacks of insanity.

Dr. Fletcher Beach said that in many cases of neurasthenia and brain fag little could be done unless sleep could be procured, and for this purpose he recommended the use of sulphonal, but he thought that it was wrong to give narcotics continually for weeks. He had not found that sulphonal gave rise to indigestion or emaciation. Dr. Percy Smith, however, said that in some cases he found it give rise to indigestion and hæmatoporphyrinuria. Dr. Percy Smith also urged that patients who could not sleep at night should be allowed to sleep whenever they felt inclined to do so, and in regard to the drug treatment of insomnia said that he thought general practitioners had not yet fully realised that other drugs would produce sleep without the very bad effects of morphia. Take the discussion as a whole, its drift seemed to be in the direction of a judicious and careful use of narcotics, but as Dr. Julius Mickle said, they should be given with brains, *i.e.*, the medical man should make a careful estimate of each particular case before giving any drug.

